# Clay Mineral Minerals as a Strategy for Biomolecule Incorporation: Amino Acids Approach

**DOI:** 10.3390/ma15010064

**Published:** 2021-12-22

**Authors:** Luciano C. Brandão-Lima, Fabrícia C. Silva, Paulo V. C. G. Costa, Edgar A. Alves-Júnior, César Viseras, Josy A. Osajima, Leilson R. Bezerra, Jose F. P. de Moura, Aline G. de A. Silva, Maria G. Fonseca, Edson C. Silva-Filho

**Affiliations:** 1 Interdisciplinary Laboratory for Advanced Materials–LIMAV, Federal Unviersity of Piauí, Teresina 64049-550, Brazil; luciano.lima@ufpi.edu.br (L.C.B.-L.); paulopvcgc@gmail.com (P.V.C.G.C.); edgar@ufpi.edu.br (E.A.A.-J.); josyosajima@ufpi.edu.br (J.A.O.); 2 Campus Senador Helvídio Nunes Barros, CSHNB, Federal Unviersityof Piauí, Picos 64600-000, Brazil; fabriciacastro@ufpi.edu.br; 3 Department of Pharmacy and Pharmaceutical Technology, University of Granada, 18071 Granada, Spain; cviseras@ugr.es; 4 Department of Animal Science, Federal University of Campina Grande, Avenida Universitária, s/n-Jatobá, Patos 58708-110, Brazil; jose.fabio@ufcg.edu.br (J.F.P.d.M.); alinegomesandrade057@gmail.com (A.G.d.A.S.); 5 Núcleo de Pesquisa e Extensão de Combustíveis e de Materiais (NPE-LACOM), Federal University of Paraíba–UFPB, João Pessoa 58051-900, Brazil; mgardennia@quimica.ufpb.br

**Keywords:** sepiolite, montmorillonite, adsorption, L-lysine, L-methionine, L-tryptophan, nutrition, ruminants

## Abstract

The potential use of amino acids by ruminal microorganisms converting them into microbial protein for ruminants makes it challenging to supplement these nutrients in an accessible form in animals’ diets. Several strategies to protect amino acids from ruminal degradation were reported, producing amino acids available for the protein used in the intestine called “bypass.” The intercalation of biomolecules in clay mineral minerals has gained notoriety due to its ability to support, protect, transport, physicochemical properties and non-toxicity. This study aimed to investigate the incorporation of L-lysine (Lys), L-methionine (Met), and L-tryptophan (Trp) amino acids in the clay minerals sepiolite (Sep) and Veegum^®^ (Veg) using the adsorption method. The characterization techniques of X-ray diffraction and infrared spectroscopy indicated the presence of biomolecules in the inorganic matrices. Elemental and thermal analyzes monitored the percentages of incorporated amino acids. They showed better incorporation capacities for Veg, such as Met-Veg < Lys-Veg < Trp-Veg and Lys-Sep < Met-Sep < Trp-Sep for sepiolite, except for the incorporation of Met. Matrices provide a promising alternative for planning the administration of biomolecules, using essential amino acids as models, and may offer an alternative to improve functional diet strategies.

## 1. Introduction

Proteins are nitrogenous organic nutrients present in all living cells and are essential to every animal’s life. The concept of ideal protein is widely used for poultry and swine, as it defines the exact balance of amino acids that can provide, without excess or lack, the requirements of all necessary amino acids and thus make it possible to express the maximum growth potential of the animal, improving feed conversion and reducing dietary costs [1,2,3]. However, for ruminant animals, amino acid supplementation should not be provided freely in the diet, as they will be degraded by ruminal microorganisms in the formation of microbial protein, providing between 70 and 80% of the metabolizable protein of these animals [4,5]. The protein requirement of ruminants is not fully satisfied by the microbial protein, requiring the passage of amino acids to the small intestine to absorb the rest of the protein demand of ruminants, known as “bypass” protein. Some chemical and/or physical processes can be used to “protect” the amino acids from ruminal degradation, providing “bypass” protein, that is, not degraded in the rumen and digested in the intestine, thus enabling a superior supply of amino acids in the intestine, improving the animal’s performance [6,7].

Clay minerals have been used in the biomaterials area as a support for the transport of biologically active molecules due to their excellent chemical, physical, and non-toxic characteristics [8,9,10,11]. A wide range of biologically active molecules has been used in systems with clay minerals as carriers, including doxorubicin [12], diclofenac [13], ketoprofen [14], and trimetazidine [15]. Amino acids have a broad spectrum of chemical reactivity, are involved in several biochemical processes, and present the possibility of modulating their interactions with solid surfaces, for example, pH, ionic strength, and others [16].

The incorporation of amino acids in inorganic matrices, such as clay minerals, occurs through the interaction between amino acids and the surface or through intercalation on structural layers. Current research aims to elucidate the exchanges and identify the factors that influence the possible reactions when these organic molecules are confined, which are interesting in different approaches [10,17,18,19].

Products intended to prepare protectants either in the form of excipients or active ingredients must feature a series of requirements for safety, stability, and high chemical inertness. In the pharmaceutical industry, clay minerals are known for being chemically and microbiologically innocuous, as well as their physical attributes, such as flavor and color, which affect acceptance by the organism, and their texture and water content, which affect technical processes [20].

Smectite and fibrous clay minerals (such as montmorillonite and sepiolite) are the most widely used in the pharmaceutical industry and bone tissue engineering because they are chemically and microbiologically innocuous [21,22,23]. 

The properties of Veegum^®^ (which is a pharmaceutical-grade clay mineral that consists mainly of montmorillonite) and sepiolite can be ideal for use as supports to incorporate amino acid molecules with the same success that has been used for pharmaceutical excipients or as active ingredients [24,25,26]. 

This study aimed to investigate the incorporation of essential amino acids in two different types of clay minerals, fibrous (sepiolite) and lamellar (Veegum^®^), as a strategy to protect biomolecules for nutritional requirements.

## 2. Materials and Methods

### 2.1. Materials

Pharmaceutical-grade Sepiolite clay mineral (Sep) from Vicálvaro (Madrid, Spain), kindly donated by TOLSA S.A. Veegum^®^ clay mineral (Veg) (Veegum HS^®^, VHS) was purchased from Vanderbilt Company (Norwalk, CT, USA). The amino acids were L-lysine 99% (Ajinomoto), L-methionine 99% (MetAMINO) and L-tryptophan 98% (Ajinomoto). Amino acids solutions were prepared with deionized water.

### 2.2. Amino Acids Incorporation

The amino acids were incorporated in clay minerals by adsorption. The amount of 1.0 g of sepiolite (Sep) or Veegum^®^ (Veg) was suspended in 30.0 mL of an aqueous solution of the amino acid (concentration of 0.01 mol L^−1^). The amino acids used were: L-lysine (Lys), L-methionine (Met) or L-tryptophan (Trp) (pKa and IP displayed in Table 1). The experiment was performed in triplicate. The suspensions were kept at 37 ± 1 °C for 48 h under constant stirring. Next, the solids were filtrated and dried at 50 °C for 24 h in an oven. The pH of the solutions was measured before and after the adsorption processes. The samples after encapsulation were named Lys-Sep, Met-Sep, Trp-Sep and Lis-Veg, Met-Veg, Trp-Veg for the incorporation of L-lysine (Lys), L-Methionine (Met), or L-tryptophan (Trp) in sepiolite (Sep), and Veegum^®^ (Veg), respectively.

### 2.3. Characterizations

The percentages of carbon and nitrogen were analyzed in a Perkin Elmer elemental analyzer, model PE-2400.

The X-ray Diffraction (XRD) was recorded using anXRD-6000 Shimadzu Ray diffractometer with CuKα radiation (λ = 1.5405 Å). XRD patterns were performed between 4 and 70° (2θ) with a step size of 0.02° and scan rate of 2 degrees min^−1^. 

Infrared analyses were performed on the Agilent Cary 630 FTIR spectrometer, using the Agilent Diamond Reflectance Total Reflectance (ATR) technique mode, with spectral resolution > 2 cm^−1^ and 32 scans. The spectra were acquired using Microlab FTIR Software (Agilent Technologies) between 4000 and 650 cm^−1^. 

Thermogravimetric analyses (TG/DTG) were performed using a TA Instrument SDT Q600 analyzer with 6.0 mg of sample and a heating rate of 5 °C min^−1^ from 25 to 1000 °C, under a nitrogen flow of 10 mL min^−1^ and using alumina crucible. 

The reproducibility of the synthesis was checked by monitoring pH and CHN for synthesized samples in triplicate. Other characterizations (XRD, FTIR and DTG) were performed for one sample in each system considering the good reproducibility of the CHN results obtained in the triplicate synthesis.

## 3. Results and Discussion

Table 2 presents the pH measurements of the amino acid solutions before and after contact with clay minerals. The data indicate that the predominant structures for the amino acids were the respective zwitterion forms even after the adsorption process, that is, presenting the respective protonated amino groups (-NH_3_^+^) and the deprotonated carboxyl groups (-COO^−^). All the pH values were between the pK_1_ and pK_2_ values of the respective amino acids, implying a positive residual charge for the L-lysine molecule (because it has two -NH_3_^+^ groups for one -COO^−^ group) and neutral for the other amino acids.

**Figure 1:**
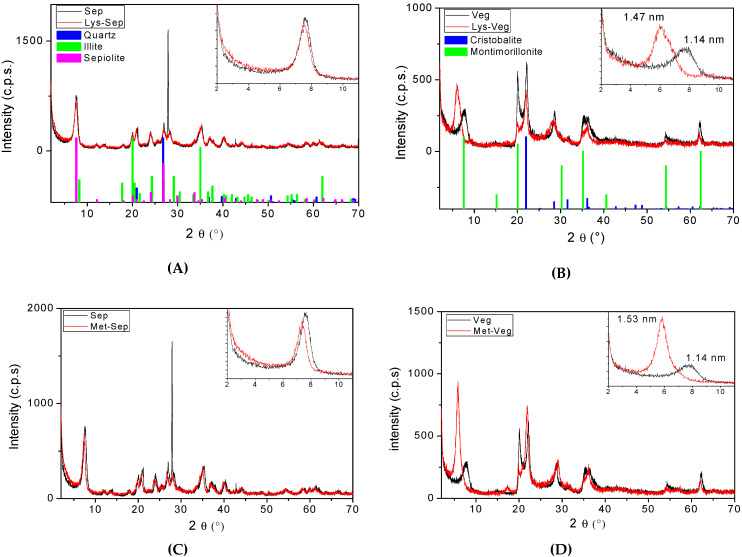

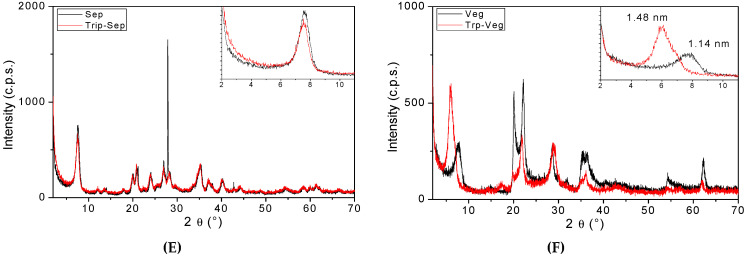
X-ray diffractograms for Sep and Veg clay mineral before and after adsorption incorporation of (**A**,**B**) L-lysine; (**C**,**D**) L-methionine; (**E**,**F**) L-tryptophan. The respective diffraction patterns for the JCPDS indexed to each diffractogram are shown in (**A**,**B**).

The characterization by X-ray diffraction (Figure 1) shows that the fibrous clay mineral (Sep) consisted of the sepiolite, quartz, and illite phases, indexed in the JCPDS cards 00-029-0863, 01-078-1257, and 00-029-1496, respectively (Figure 1A). On the other hand, in the diffractograms of the lamellar clay mineral (Veg) (Figure 1B), the characteristic reflections are indexed to the phases of montmorilonite (JCPDS 00-002-0037), and cristobalite (JCPDS 01-082-1409).

Different behaviors followed after the amino acid incorporation process depending on each type of clay mineral studied. The non-expandable characteristic of the Sep fibrous clay mineral may explain the non-existence of expressive changes in the diffraction patterns. The only observable difference was a slight decrease in the relative intensity of reflection 011, which appears approximately at 7.3°, as seen in the respective magnifications (Figure 1A,C,E). It suggests that the interactions between organic molecules on the surface or within the clay mineral channels induced a decrease in the stacking order, without significant changes in the crystal structure and organization [20,23]. 

In the diffractograms that represent the interaction of Veg with amino acids (Figure 1B,D,F), shifts in reflections relative to the basal spacing were observed after the amino acid–clay mineral interactions. In the starting material, it appears at approximately 7.7°, shifting for lower angles, that is, increasing the basal spacing from 1.14 nm in Veg to 1.47 nm, 1.53 nm, and 1.48 nm for the incorporation by the adsorption of lysine (Lys-Veg), methionine (Met-Veg), and tryptophan (Trp-Veg), respectively.

**Figure 2:**
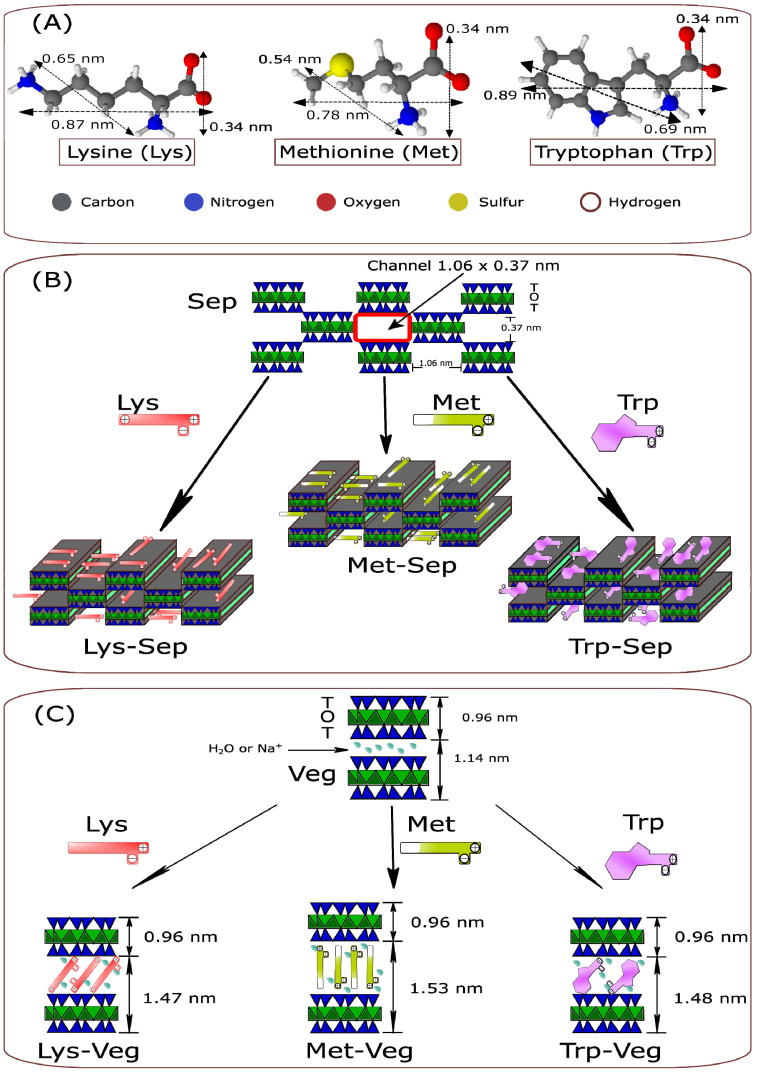
(**A**) Schematic illustration for molecular structures of amino acids and their dimensions (calculated using Chem Sketch 12.0 software (ACD/Labs, Toronto, ON, Canada)). Proposals for the incorporation of amino acids in (**B**) Sep and (**C**) Veg.

Figure 2A displays the structures of the amino acids and their respective dimensions. The dimensions of all the incorporated molecules were compatible with the size of the Sep channels, which followed their varied three-dimensional arrangements and permeated the three-dimensional ducts of the fibrous clay mineral crystal structure, as observed in Figure 2B.

The thickness of the Veg layer was 0.96 nm, and the interlamellar spacing was 0.51 nm, 0.57 nm, and 0.52 nm in Lys-Veg, Met-Veg, and Trp-Veg, respectively. Due to the dimensions of the respective molecules of the amino acids incorporated (Figure 2A), there were two arrangements in the space between clay mineral layers that can propose. First, the molecules displayed an inclined orientation relative to the silicate interlayer surface (compatible with the dimensions of Lys and Trp); and second, the amino acid featured a perpendicular direction (consistent with the sizes of the Met). In both cases, the amino acids form a monolayer structure with the positively charged side chain amino groups pointing towards the surface [18], as shown in Figure 2C.

**Figure 3:**
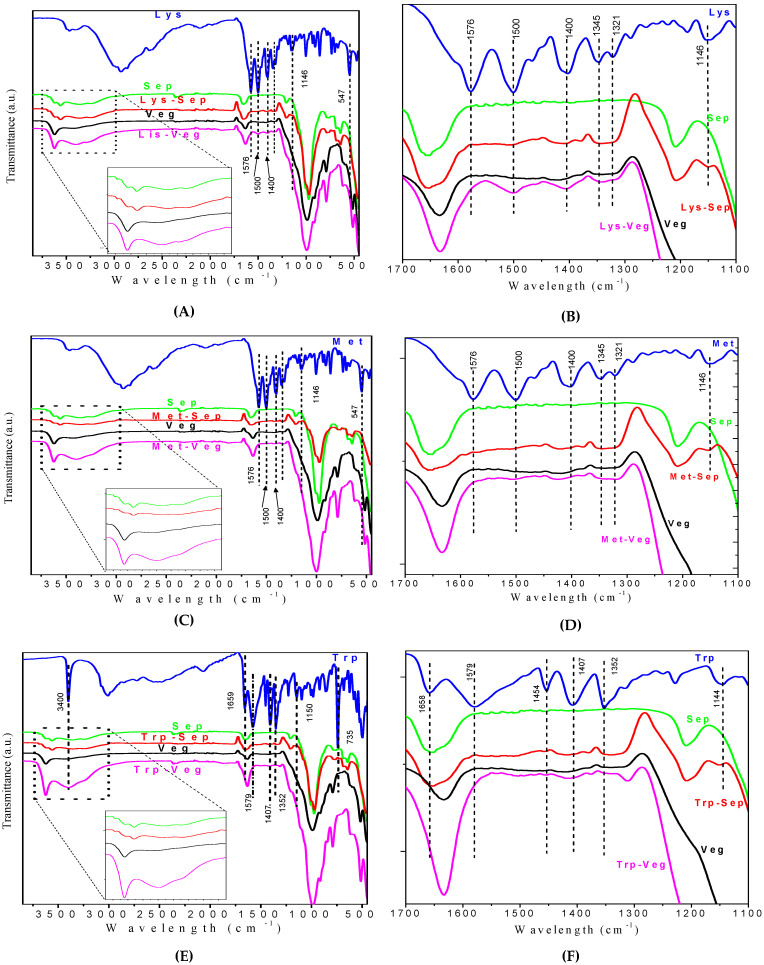
FTIR spectra of samples before and after incorporation of (**A**,**B**) lysine, (**C**,**D**) methionine, and (**E**,**F**) tryptophan accompanied by the spectra of the respective amino acids.

Figure 3 shows the IR spectra referring to Sep and Veg before the incorporation of the respective amino acids, which exhibited characteristic bands in similar regions but showed differences in intensities and bandwidths. The bands in regions between 3750–3150 cm^−1^ and at 1650 cm^−1^ were attributed to the stretching and deformation modes of structural hydroxyl groups and/or intercalated water molecules or constituents of the structure, respectively [24,25,26,27,28]. Absorption bands related to Si-O-Si tetrahedral sheets appear at 1250–950 cm^−1^, and to M-OH octahedral sheets appear in the low-wavelength region (M indicates a metal, for example, Al–Al–OH, Mg–Mg–OH, Al–Fe–OH) [24,29,30,31].

The spectra referring to amino acids not adsorbed on clay minerals presented bands referring to the functional groups characteristic of biomolecules. In the L-lysine range (Figure 3A,B), the broadband in the region between 3300–2500 cm^−1^ presented contributions from the symmetrical and asymmetrical stretches of the N-H and C-H groups. The band between 2200–2000 cm^−1^ suggested the presence of protonated groups, which can be attributed to the combination of the δNH_3_^+^ and τNH_3_^+^ modes. The bands 1576 cm^−1^ and 1400 cm^−1^ were attributed to the antisymmetric and symmetric stretches of the carboxylate group (COO-). The band at 547 cm^−1^ represented the symmetric angular deformation δs(O-C-O) of this group. The deformation bands of NH_3_^+^ and CH_2_ groups appeared at 1500 cm^−1^ and the region between 1370–1300 cm^−1^, respectively [28,32,33].

The L-methionine molecule is similar to the L-lysine molecule, except for the C-S-C bond and the presence of only one amino group. These characteristics are not easily detectable by FTIR (see Figure 3C,D). The C–S bond usually features weak bands and may overlap. Due to this limitation, there is similarity between the spectra of these amino acids, considering the same attributions mentioned for L-lysine [34,35].

In the spectra of L-tryptophan (Trp in Figure 3E,F), the stretch mode of the N-H bond of the indole group was observed as a narrow and intense band at 3400 cm^−1^. The bands in the region between 3280–2500 cm^−1^ were attributed to stretches of aliphatic and aromatic C-H groups and the vibrations of the NH_3_+ group characteristic of the zwitterion form. The angular deformations δNH_3_^+^, δCH_2_ and ρNH_3_^+^ appeared at 1579 cm^−1^, 1454 cm^−1^, and 1144 cm^−1^, respectively. For the carboxylate group, the vibration modes ν_as_COO^−^ and ν_s_COO^−^ were observed at 1658 cm^−1^ and 1407 cm^−1^, respectively. The deformation mode δ_s_(O-C-O) appeared at 735 cm^−1^. The band at 1352 cm^−1^ was attributed to the vibration ν(C-N) of the indole group [36,37,38,39].

The spectra in Figure 3 represent the clay minerals after incorporating amino acids and present the predominance of the vibrational modes of the respective inorganic matrix. However, some alterations can be observed in the OH stretch and deformation bands, which were broadened and more intense than Sep and Veg. It can indicate intermolecular amino acid/clay mineral interactions and contributions by overlapping modes in amino acids. The emergence of new signals (mainly in the range between 1700–1100 cm^−1^, as shown in Figure 3B,D,F) indicate successful amino acid incorporation [24,33].

**Figure 4:**
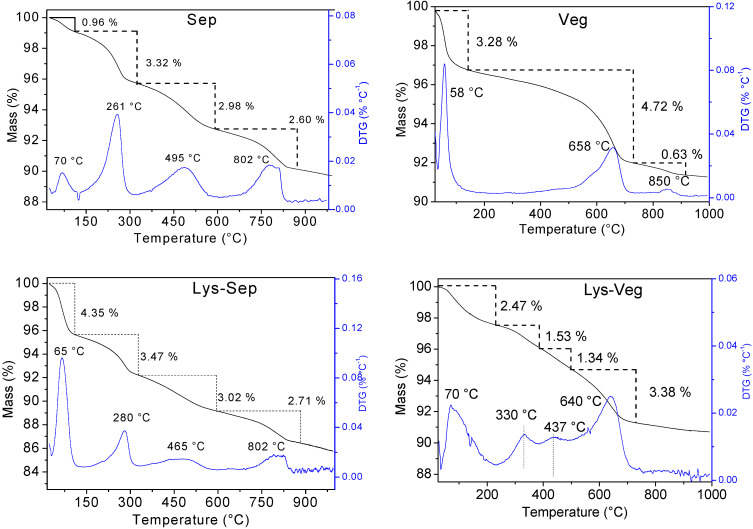

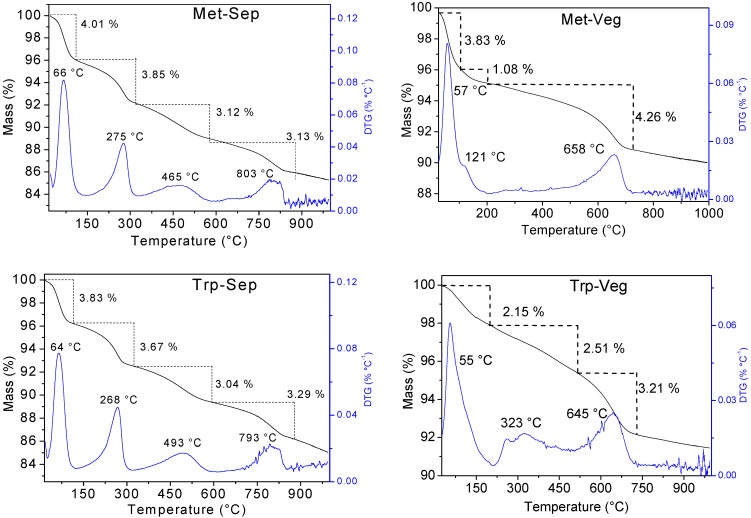
TG/DTG curves of clay minerals before and after amino acids incorporations.

Figure 4 illustrates the TG/DTG curves for Sep and Veg before and after amino acid incorporation.

The Sep TG curve and derivative (DTG) presented four mass loss events with maximum temperatures (Tmax) at 70, 261, 495, and 802 °C and percentages of mass loss equal to 0.96; 3.32; 2.98 and 2.60%, respectively. The first event was associated with the elimination of water molecules superficially adsorbed on the clay mineral structure. The second was attributed to the loss of interchannel zeolitic water molecules and hydrogen bonds in the fibrous structure. Finally, the last two events were attributed to clay mineral dehydroxylation [29,40].

The three mass loss events observed in the TG/DTG curve of the Veg lamellar clay mineral present Tmax at 58, 658, and 850 °C. The first event was attributed to the elimination of surface water and a loss percentage of 3.28%. The last events were associated with eliminating coordinated water firmly bound with the octahedral sheet and dehydroxylation from the silanol groups (4.72 and 0.62%, respectively). The total mass loss was 5.34% [24,41,42].

After the incorporation of amino acids into Sep, the degradation events were maintained. However, organic molecules on the surface or inside the clay mineral channels altered the intermolecular interactions with the physisorbed water molecules and interchannel. This promoted small changes in temperatures and percentages of mass loss and changes indicative of adsorbed amino acids on the surface or within the fibrous clay mineral channels. In the case of amino acid incorporation in Veg, new thermal degradation events occurred in the range between 120–550 °C, which was attributed to the mass losses of the respective incorporated organic matter.

Table 3 serves to identify the differences observed in each degradation event, comparing the temperatures and respective percentages of loss, where an important observation concerns the increase in the percentage of loss of masses, especially in events from 200–550 °C, for all profiles compared to Sep. This can be attributed to the contributions of organic matter loss related to the respective incorporated amino acids plus water loss and clay mineral dehydroxylation [24].

The results of the elemental analysis for carbon and nitrogen are displayed in Table 4. Considering that clay minerals do not feature nitrogen in their composition, the percentages of nitrogen obtained in the samples after adsorption can be associated with the contribution of the incorporated amino acid molecules. Therefore, the presence of the organic molecules in the clay samples indicated successful reactions. The experimental C/N ratios were close to the respective C/N ratios expected from the molecular formulas of the amino acids, indicating that the incorporation of the amino acids occurred without structural changes or combinations resulting from possible reactions during the adsorption process.

The Sep and Veg precursor clay minerals featured minimal carbon content, which was in the instrument error. The total percentage of carbon calculated in the samples after adsorption was associated with the respective incorporated amino acid. The percentage carbon values could estimate the amount of amino acid contained from the expected percentage of carbon from the molar mass of the individual amino acid. The CHN data were also used to check the reproductibilty of the syhthesis. Figure S1 illustrates the results of the amounts of amino acids incorporated by CHN in each system and suggests that the procedure was reproducible considering the standard deviations. No significant differences were observed between the obtained results with three measurements for one sample or three measurements for the three samples of the triplicate. 

From the results of the thermal analysis, it was also possible to estimate the amounts of amino acids incorporated. In the case of Veg clay mineral, the values were easily obtained from the percentages of mass loss in new thermal degradation events that appeared in the range between 120–550 °C. In the case of the incorporation of amino acids in Sep, the estimates were calculated by differences in percentage residues before and after the incorporation of the respective amino acid. The percentage residuals were calculated disregarding the losses of physisorbed water, since the superpositions of thermal degradations of organic molecules with events in clay mineral make impossible the use of the same approach for layered clay mineral. The results obtained with both characterization techniques are shown in Table 5. The results of the amounts of the incorporated amino acid for both measurements were in concordance in the smectite sample. For the sepiolite systems, the analysis showed a substantial difference considering the occurrence of simultaneous mass losses in the same temperature range, especially for Lys-Sep. However, TG was useful for comparing different modified samples obtained with the same clay mineral.

The Sep clay mineral presented the incorporation capacity order Lys < Met < Trp and the Veg clay mineral presented the order Met < Trp < Lys, regardless of the technique used for the calculation. The Lys-Sep was the only mineral to present significantly different values when comparing the estimates obtained in each method, in which the most significant error when calculating the percentage of Lys in the sample by TG/DTG may have been associated with the fact that it was the most hydrophilic amino acid, causing stronger interactions with molecules of water physisorbed in the interchannel.

Incorporating amino acids into Sep clay mineral occurred with electrostatic interactions between the positively charged -NH_3_^+^ groups of organic molecules and the residual negative charge present on the clay mineral surface. In the specific case of tryptophan, the aromatic rings present in the structure provided the additional possibility of interactions such as Van der Waals force and the possibility of forming stacks in multiple layers between the plane structures attracted by such forces. This was possibly responsible for the greater capacity of incorporation of Trp on the surface of Sep.

In the case of the smectite clay mineral Veg, the incorporations occurred through the exchange between the Na^+^ ions present in the clay mineral’s interlamellar space and the organic molecules. As described above, the Lys molecule featured a positive residual charge at the pH studied because it included two -NH_3_^+^ groups, facilitating the cation exchange process, which may explain the more significant amount of Lys incorporated. The other amino acids featured a neutral residual charge, leading to the false impression that it was more difficult to accommodate the Trp molecules than the Met due to their larger size and volume. However, the additional possibility of Van der Waals force-type interactions with the π bonds of aromatic rings justifies the more significant amount of Trp incorporated when compared to Met.

## 4. Conclusions

Using the adsorption method, it was possible to incorporate molecules of the amino acids L-lysine, L-methionine, and L-tryptophan into sepiolite and Veegum^®^ clay minerals, and the characterization techniques confirmed these incorporations. Two different behaviors were found by XRD, in which the fibrous clay mineral Sep suggested a decrease in the stacking order due to the presence of amino acids incorporated on the surface or inside the channels, while the expansion of the clay mineral Veg showed changes in the respective peaks referring to the reflection d_001_, which suggested increases in the basal spacing from 11.45 Å in Veg to 14.75 Å, 15.34 Å, and 14.80 Å for the incorporation of lysine (Lys-Veg), methionine (Met-Veg), and tryptophan (Trp-Veg), respectively. The results obtained by FTIR support the presence of the respective functional groups incorporated into the clay minerals. The values for the amounts of incorporated amino acids were obtained from the elemental analysis; the percentages of mass loss from the thermal analysis results followed similar tendencies and point to the greater incorporation capacity of Veegum^®^, with the exception of t cases of methionine incorporation, obtaining the orders of incorporation capacity Lys-Sep < Met-Sep < Trp-Sep for sepiolite and Met-Veg < Trp-Veg< Lys-Veg for Veegum^®^. Thus, these results provide a promising alternative for planning the administration of biomolecules, tested in this work with essential amino acids, and their continuity may offer an option to improve administration strategies in available diets, as well as to reduce feeding costs by providing matrices that are low-cost and that feature relatively easy production.

## Figures and Tables

**Table 1:** The pka and isoelectric point values of lysine, methionine, and tryptophan.

Amino Acid	pK_a_ Values			^1^ IP
	pK_1_ (-COOH)	pK_2_ (-NH_3_^+^)	pK_3_ (Grupo R)	
Lysine	2.18	8.95	10.53	9.74
Methionine	2.28	9.21	-	5.74
Tryptophan	2.38	9.39	-	5.89

^1^ Isoelectric point.

**Table 2:** pH measures of amino acid solutions before and after the adsorption process.

Sample	pH		Sample	pH	
	Before	After		Before	After
Lys-Sep	7.41 ± 0.24	7.79 ± 0.22	Lys-Veg	7.41 ± 0.24	8.30 ± 0.18
Met-Sep	7.23 ± 0.12	7.83 ± 0.13	Met-Veg	7.23 ± 0.12	7.93 ± 0.29
Trp-Sep	7.10 ± 0.09	7.76 ± 0.22	Trp-Veg	7.10 ± 0.09	8.02 ± 0.11

**Table 3:** Values of maximum temperatures and respective mass losses in each event obtained from the TG/DTG curves.

Sample	Tmax (°C)				Mass Loss (%)				Residue (%)
	1st	2nd	3rd	4th	1st	2nd	3rd	4th	
Sep	70	261	495	802	0.96	3.32	2.98	2.60	90.57
Lys-Sep	65	280	465	802	4.35	3.47	3.02	2.71	89.67
Met-Sep	66	275	465	803	4.01	3.85	3.12	3.13	88.86
Trp-Sep	64	268	493	793	3.83	3.67	3.04	3.29	88.44
Veg	58	658	850	-	3.28	4.72	0.62	-	94.37
Lys-Veg	70	330	437	640	2.47	1.53	1.34	3.38	92.95
Met-Veg	57	121	658	-	3.83	1.08	4.26	-	93.57
Trp-Veg	55	323	645	-	2.15	2.51	3.21	-	93.34

**Table 4:** Results obtained from elemental analysis (CHN) for the obtained samples.

Sample	(%)		(mmol g^−1^)		C/N Theoretical	C/N Experimental
	C	N	C	N		
Sep	0.09 ± 0.02	-	0.07 ± 0.02	-	-	-
Lys-Sep	0.78 ± 0.03	0.24 ± 0.01	0.65 ± 0.03	0.17 ± 0.01	3.00	3.82
Met-Sep	0.67 ± 0.04	0.14 ± 0.01	0.56 ± 0.04	0.11 ± 0.01	5.00	5.09
Trp-Sep	1.72 ± 0.09	0.39 ± 0.02	1.43 ± 0.09	0.28 ± 0.02	5.50	5.15
Veg	0.08 ± 0.01	-	0.06 ± 0.01	-	-	-
Lys-Veg	1.42 ± 0.07	0.53 ± 0.03	1.18 ± 0.07	0.38 ± 0.03	3.00	3.10
Met-Veg	0.45 ± 0.02	0.12 ± 0.01	0.38 ± 0.02	0.08 ± 0.01	5.00	4.75
Trp-Veg	1.83 ± 0.09	0.40 ± 0.02	1.52 ± 0.09	0.29 ± 0.02	5.50	5.24

**Table 5:** Amounts of incorporated amino acid obtained from elemental analysis and thermal analysis data.

Sample	Incorporated Amino Acid (%)		Sample	Incorporated Amino Acid (%)	
	Elemental Analysis	TG/DTG		Elemental Analysis	TG/DTG
Lys-Sep	1.58 ± 0.03	0.90	Lys-Veg	2.88 ± 0.07	2.87
Met-Sep	1.66 ± 0.04	1.71	Met-Veg	1.12 ± 0.02	1.08
Trp-Sep	2.66 ± 0.09	2.13	Trp-Veg	2.83 ± 0.09	2.51

## Data Availability

Not applicable.

## Supplementary Materials

The following are available online at https://www.mdpi.com/article/10.3390/ma15010064/s1, Figure S1: Amount of incorporated amino acids obtained from triplicate % C measures of four adsorption procedure replicates, to proof the reliability of the method.
