# Supplementary material for: Clay Mineral Minerals as a Strategy for Biomolecule Incorporation: Amino Acids Approach
Source: Materials (Basel). 2021 Dec 22;15(1):64. doi: 10.3390/ma15010064 (PMC8745852; doi:10.3390/ma15010064)
Supplement: Supplementary file 1 [file materials-15-00064-s001.zip › materials-1498171-supplementary.pdf]

Clay mineral minerals as a strategy for biomolecules incorporation: amino acids approach

Luciano C. Brandão-Lima[1], Fabrícia C. Silva[2], Paulo V. C. G. Costa[1], Edgar A. Alves-Júnior[1], Cesar Viseras[3], Josy A. Osajima[1], Leilson R. Bezerra[4], Jose F. P. de Moura[4], Aline G. de A. Silva[4], Maria G. Fonseca[5], Edson C. Silva-Filho[1,*]

Supplementar Material

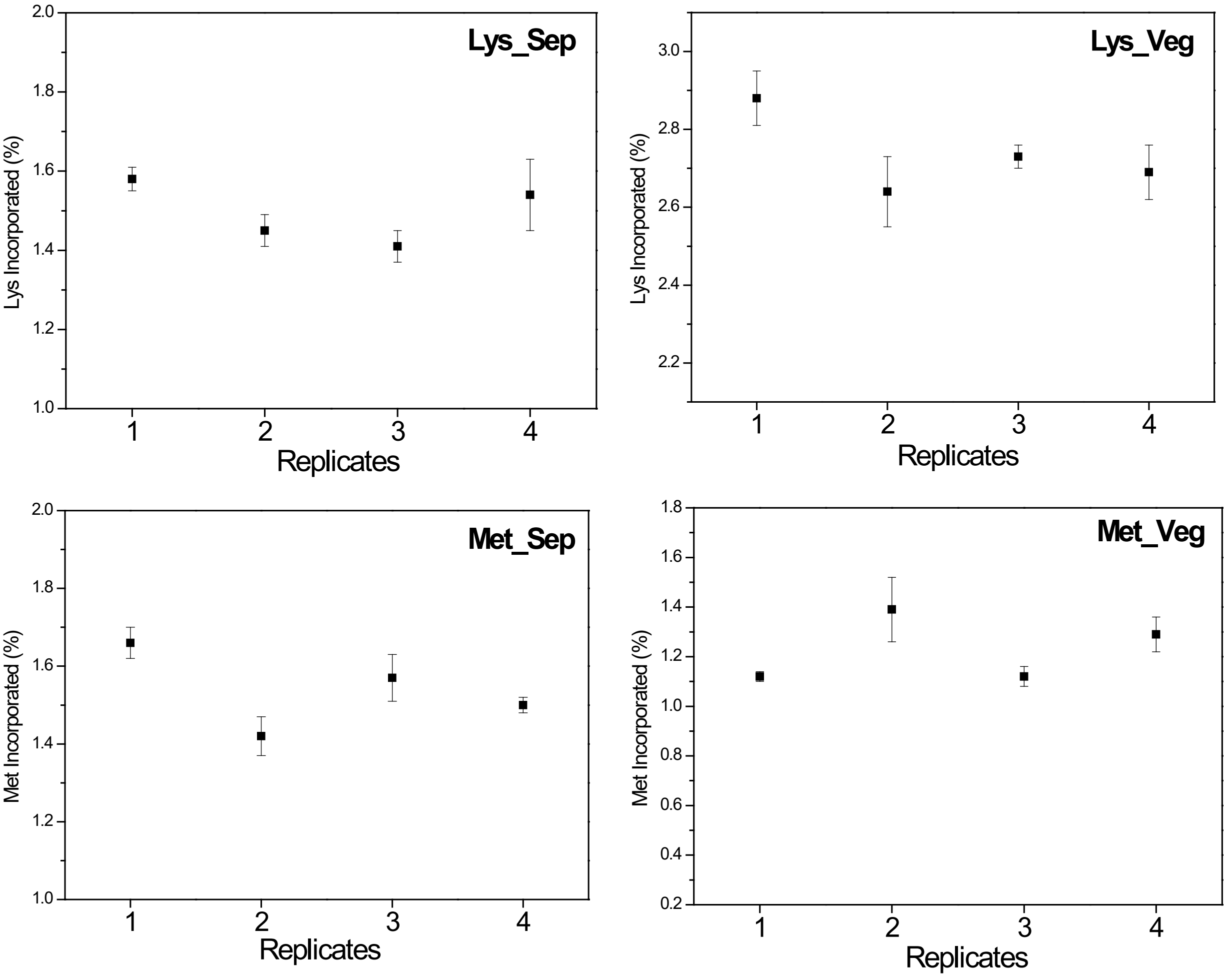

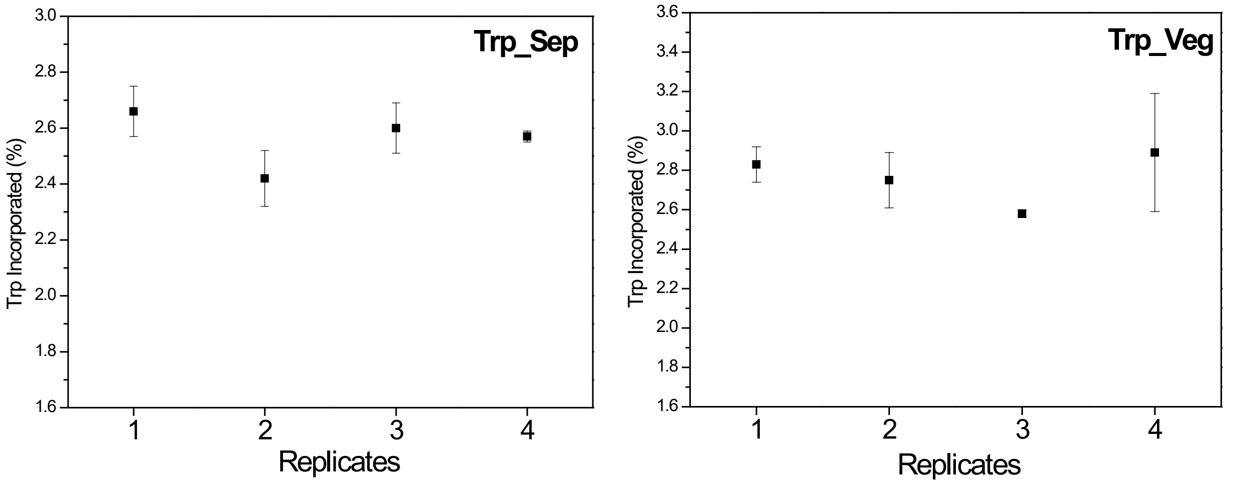

Figure S1. Amount of incorporated amino acids obtained from triplicate % C measures of four adsorption procedure replicates, to proof the reliability of the method.
